# Telomere extension by telomerase and ALT generates variant repeats by mechanistically distinct processes

**DOI:** 10.1093/nar/gkt1117

**Published:** 2013-11-12

**Authors:** Michael Lee, Mark Hills, Dimitri Conomos, Michael D. Stutz, Rebecca A. Dagg, Loretta M.S. Lau, Roger R. Reddel, Hilda A. Pickett

**Affiliations:** ^1^Telomere Length Regulation Group, Children’s Medical Research Institute, Westmead NSW 2145, Australia, ^2^Cancer Research Unit, Children’s Medical Research Institute, Westmead NSW 2145, Australia, ^3^Terry Fox Laboratory, BC Cancer Agency, Vancouver V5Z 1L3, Canada, ^4^Sydney Medical School, University of Sydney, Sydney NSW 2006, Australia and ^5^Children’s Cancer Research Unit, The Children’s Hospital at Westmead, Westmead NSW 2145, Australia

## Abstract

Telomeres are terminal repetitive DNA sequences on chromosomes, and are considered to comprise almost exclusively hexameric TTAGGG repeats. We have evaluated telomere sequence content in human cells using whole-genome sequencing followed by telomere read extraction in a panel of mortal cell strains and immortal cell lines. We identified a wide range of telomere variant repeats in human cells, and found evidence that variant repeats are generated by mechanistically distinct processes during telomerase- and ALT-mediated telomere lengthening. Telomerase-mediated telomere extension resulted in biased repeat synthesis of variant repeats that differed from the canonical sequence at positions 1 and 3, but not at positions 2, 4, 5 or 6. This indicates that telomerase is most likely an error-prone reverse transcriptase that misincorporates nucleotides at specific positions on the telomerase RNA template. In contrast, cell lines that use the ALT pathway contained a large range of variant repeats that varied greatly between lines. This is consistent with variant repeats spreading from proximal telomeric regions throughout telomeres in a stochastic manner by recombination-mediated templating of DNA synthesis. The presence of unexpectedly large numbers of variant repeats in cells utilizing either telomere maintenance mechanism suggests a conserved role for variant sequences at human telomeres.

## INTRODUCTION

Human telomeres are composed of double-stranded (ds) repeat arrays of a TTAGGG repeat unit terminating in a single-stranded (ss) G-rich overhang (1–4). The fidelity of the TTAGGG sequence is thought to be maintained by the action of the ribonucleoprotein enzyme telomerase, which uses an intrinsic RNA molecule (hTR) containing the CAAUCCCAAUC template region and the reverse transcriptase component (hTERT), to synthesize telomeric DNA *de novo* onto the chromosome terminus (5–7). Consequently, telomerase both compensates for telomere erosion during cell proliferation and conserves the telomeric sequence identity.

In humans, telomerase is active in the germline, early in embryogenesis, in stem cells, and following stimulation of an immune response (8,9). In these circumstances telomerase is stringently regulated to enable increased cellular proliferation without permitting tumorigenic escape. Loss of this stringent regulation may result in upregulation of telomerase activity to a level that is sufficient to prevent telomere shortening and permit unlimited proliferation (i.e. cellular immortalization), which is a key step in tumorigenesis. While the great majority of tumors activate telomerase, a smaller proportion of tumors prevent telomere shortening by an alternative lengthening of telomeres (ALT) pathway (10), which involves homologous recombination-mediated telomere extension using telomeric DNA as the template (11).

Telomerase adds DNA to the distal termini of telomeres, whereas the proximal regions contain degenerate repeats, such as TTGGGG, TGAGGG and TCAGGG (12,13). Telomere variant repeat mapping by PCR (TVR-PCR) has enabled characterization of variant repeat sequences in the proximal regions of specific telomeres (14–17). However, both the range and the extent of sequence divergence have remained elusive due in large part to technical difficulties in sequencing repetitive DNA. It is feasible that extensive degenerate repeats extend to the boundary at which telomerase exerts an effect, however this boundary is likely to be subject to substantial variation arising from telomere attrition, stochastic telomere rapid deletion events and telomere lengthening following activation of a telomere maintenance mechanism (TMM).

Numerous studies have addressed the effects of variant repeats by exogenously expressing a mutant hTR with an altered template region, which directs the incorporation of mutant repeats at the chromosome terminus. Telomerase-mediated variant repeat incorporation is detrimental, and has been reported to decrease cell proliferation and viability, increase cellular senescence and apoptosis (18–21), cause chromosome fusions and the presence of micronuclei (22–24), sensitize cells to chemotherapeutic agents (25) and increase telomeric recombination (26). It has been proposed that these phenotypes result from decreased binding of the telomere-specific protein complex shelterin, which comprises TRF1, TRF2, POT1, TPP1, TIN2 and RAP1 (27). TRF1 and TRF2 specifically bind to the ds canonical telomere repeats and display reduced binding affinities to variant repeats (16,24). We have previously demonstrated that telomerase-mediated incorporation of TCAGGG variant repeats results in sequence-specific binding of a group of orphan nuclear receptors, and competitive inhibition of shelterin binding (28).

The difficulties of sequencing repetitive DNA by conventional techniques have resulted in a paucity of DNA sequence information for human telomeres. In this study we use parallel whole-genome sequencing to characterize telomere sequence content in a panel of 18 mortal, telomerase-positive and ALT cell lines, in order to determine the extent and contribution of non-canonical telomere sequences to telomere biology. We demonstrate that telomere variant repeats are prevalent in all human telomeres but that there is a different pattern of telomeric content in cells where the telomeres are maintained by telomerase compared to those maintained by ALT, which implies that these TMMs generate variant repeats by different mechanisms.

## MATERIALS AND METHODS

### DNA preparation

Cell lines were cultured in DMEM (Gibco) supplemented with 10% (vol/vol) FBS in a humidified incubator at 37°C with 5% CO_2_. Cells were harvested by trypsinization, washed in PBS and resuspended in lysis solution [100 mM Tris–HCl, pH 7.5, 10 mM EDTA, 100 mM NaCl, and 1% (wt/vol) *N*-lauroylsarcosine]. Lysates were subjected to RNase A (50 μg/ml) treatment for 20 min at room temperature followed by Proteinase K (100 μg/ml) treatment for 8 h at 55°C. DNA was extracted using equal volumes of phenol/chloroform/isoamyl alcohol (25:24:1) and the resulting phases were separated by centrifugation at 1000*g* for 10 min at room temperature using Phase Lock Gel Light tubes (5 PRIME). The DNA was ethanol precipitated (0.1 vol of 3 M sodium acetate and 2.5 vol of 100% ethanol) and resuspended in TE (10 mM Tris–HCl, pH 8.0 and 1 mM EDTA).

### Whole-genome sequencing

Genomic DNA was fragmented by sonication and the fragment ends were phosphorylated and A tailed. Indexed forked adapters were then ligated, and the resultant libraries were PCR amplified and applied to two lanes of an Illumina HiSeq 2000. Results were aligned to the genome (HG18) using Burrows–Wheeler Alignment tool (29). Sequence reads were 100 nt. The telomeric subset of reads was extracted using a previously reported custom script (http://sourceforge.net/projects/motifcounter/) (28) incorporating functions from SAMtools (30) and standard Linux pattern-matching programs. Genome coverage was calculated using BEDtools. Telomere counts were normalized to genome coverage.

### Terminal restriction fragment length analysis

Terminal restriction fragments (TRFs) were separated as described previously (28). Briefly, genomic DNA was digested with HinfI and RsaI, ethanol precipitated, and 2 μg was loaded on a 1% (wt/vol) agarose gel in 0.5× TBE. Pulsed-field gels were run at 6 V for 14 h at 14°C, dried for 2 h at 60°C, denatured and subjected to in-gel hybridization overnight with a γ-[^32^P]-ATP-labeled (CCCTAA)_4_ oligonucleotide probe. Gels were washed and exposed to a PhosphorImager screen overnight.

### Telomere fluorescence *in situ* hybridization

Telomere and variant repeat fluorescence *in situ* hybridization (FISH) was carried out as described previously (28). Briefly, chromosome preparations were obtained from colcemid arrested cells by standard cytogenetic methods, as described previously (28). Slides were subjected to a graded ethanol dehydration series and allowed to air dry and overlaid with Texas red–OO-(CCCTAA)_3_ telomeric and Alexa 488–OO-(CCCTAC)_3_ PNA probes (Panagene) in PNA hybridization solution [70% (vol/vol) deionized formamide, 0.25% (vol/vol) NEN blocking reagent [PerkinElmer], 10 mM Tris–HCl, pH 7.5, 4 mM Na_2_HPO_4_, 0.5 mM citric acid and 1.25 mM MgCl_2_), denatured for 3 min at 80°C, and hybridized for 2 h at room temperature. Competing strand-specific canonical and variant repeat PNA probes were hybridized simultaneously in order to minimize repeat cross-hybridization. Slides were washed in PNA wash A [70% (vol/vol) formamide and 10 mM Tris–HCl, pH 7.5], and then in PNA wash B [50 mM Tris–HCl, pH 7.5, 150 mM NaCl and 0.08% (vol/vol) Tween 20] and DAPI at 50 ng/ml added to the final wash. Slides were mounted using DABCO [90% (vol/vol) glycerol, 2.3% (vol/vol) 1,4-diazabicyclo(2.2.2)octane (Sigma-Aldrich) and 50 mM Tris–HCl, pH 8.0]. Slides were imaged using an Axioimager M1 microscope (Carl Zeiss).

### Telomere content analysis by dot blot

Total telomeric DNA content was quantitated as described previously (28). Briefly, digested DNA (400 ng) was dot blotted onto a 2× SSC-soaked Biodyne B 0.45-μm nylon membrane (Pall). DNA was UV cross-linked onto the membrane and hybridized overnight to a γ-[^32^P]-ATP–labeled (CCCTAA)_4_ oligonucleotide probe. The membrane was washed and exposed to a PhosphorImager screen overnight.

### Telomere content analysis by quantitative PCR

Genomic DNA was diluted to 1 ng/µl. Quantitative PCR (qPCR) was carried out in triplicate to compare telomere sequence content to the single-copy gene VAV2. Each 25 µl PCR reaction contained 1× QuantiFAST SYBR Green master mix (Qiagen), 10 mM DTT, 0.5 µl dimethyl sulfoxide, 5 µl DNA template and telomere primers: 2009 telomere primers forward 300 nM 5′-ACACTAAGGTTTGGGTTTGGGTTTGGGTTTGGGTTAGTGT-3′ and reverse 400 nM 5′-TGTTAGGTATCCCTATCCCTATCCCTATCCCTATCCCTAACA-3′, 2002 telomere primers forward 300 nM 5′-GGTTTTTGAGGGTGAGGGTGAGGGTGAGGGTGAGGGT-3′ and reverse 400 nM 5′-TCCCGACTATCCCTATCCCTATCCCTATCCCTATCCCTA-3′ and VAV2 primers: forward 700 nM 5′-TGGGCATGACTGAAGATGAC-3′ and reverse 400 nM 5′-ATCTGCCCTCACCTTCTCAA-3′. PCRs were performed with the Rotor-Gene Q (Qiagen) real-time cycler for 95°C for 5 min, 33 cycles of 95°C for 15 s and 56°C for 2 min (telomeric primers), and 95°C for 5 min, 40 cycles of 95°C 15 s, 61°C 30 s and 72°C 20 s (VAV2 primers) (‘2009 method’) (31) and for 95°C for 5 min, 30 cycles of 95°C 15 s, 56°C 15 s and 72°C 60 s (‘2002 method’) (32). Serially diluted DNA standards were used to generate standard curves and Rotor-Gene Q series software was used to analyze data.

## RESULTS

### Telomere sequence content can be determined by whole-genome sequencing

Whole-genome sequencing has previously been applied to the measurement of telomeric DNA content by quantitation of telomere reads using the extraction criteria 4× TTAGGG (33). Here, we applied whole-genome sequencing to the specific analysis of telomere sequence content in a panel of mortal, telomerase-positive and ALT cell lines using the Illumina HiSeq 2000 platform (Table 1). This panel included spontaneously, chemically, or SV40-immortalized, and tumor-derived cell lines, as well as matched mortal cell strains. Telomere sequences cannot be assembled to the human genome since the multiple mapping locations on the reference sequence result in a mapping quality with a phred score of zero. For this reason we analyzed and counted reads that contained telomeric sequences in order to evaluate telomere sequence content.

**Figure 1.**
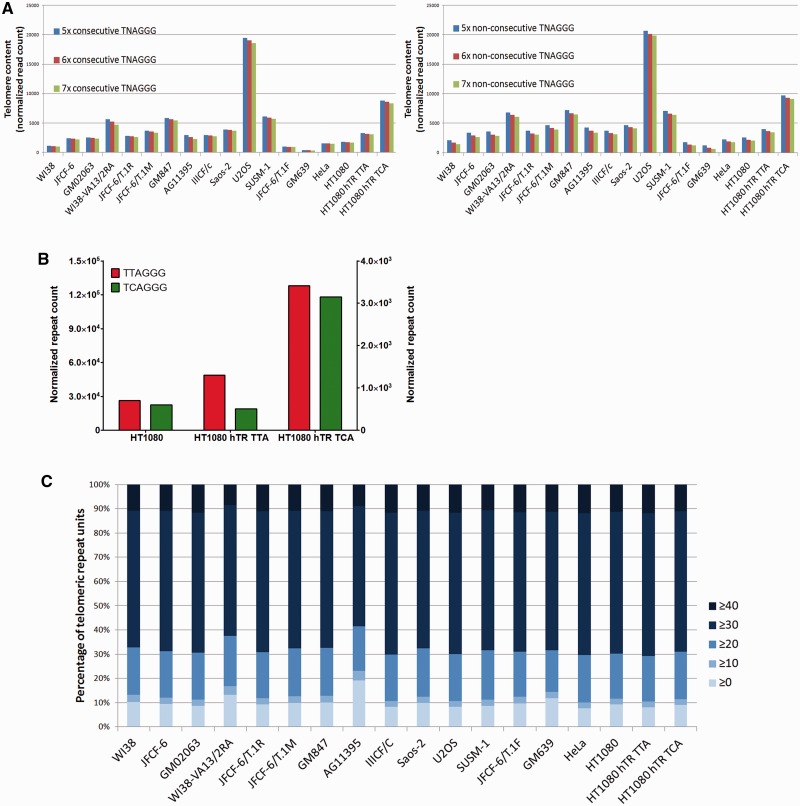
Whole-genome sequencing can be used to analyze telomere sequence content. (**A**) Telomere read count normalized to genome coverage using variable extraction criteria (5× TNAGGG, 6× TNAGGG, 7× TNAGGG, appearing consecutively, or non-consecutively). (**B**) Canonical telomere repeat count (red; left axis) and TCAGGG variant repeat count (green; right axis) in telomere extracted reads from HT1080 parental, HT1080 hTR TTA (expressing exogenous wild-type hTR) and HT1080 hTR TCA (expressing exogenous wild-type and mutant hTR encoding the TCAGGG hexamer) cell lines. (**C**) Percentage of telomeric repeat units with an average quality score of ≥40, ≥30, ≥20, ≥10 and ≥0. A quality score of 30 corresponds to the probability of an incorrect base call of 1 in 1000, and a quality score of 20 corresponds to the probability of an incorrect base call of 1 in 100.

In order to validate the technique, we compared the number of reads that were extracted using the variable selection criteria 5× TNAGGG, 6× TNAGGG and 7× TNAGGG, appearing either consecutively or non-consecutively (Figure 1A). Small differences in the number of extracted telomere reads were identified, with the less stringent criteria resulting in a slightly greater number of extracted reads, and the more stringent criteria resulting in slightly fewer extracted reads (Figure 1A). The differences were equally small in all cell lines (Figure 1A).

**Table 1.** Cell line information and sequence coverage

Cell line	Telomere maintenance mechanism	Mechanism of transformation	Total number of reads	Number of telomere reads (6× non-consecutive TNAGGG)
WI38	mortal	n/a	23870638	1146
JFCF-6	mortal	n/a	26723886	2240
GM02063	mortal	n/a	26812462	2364
JFCF-6/T.1F	telomerase	SV40	30075606	1229
GM639	telomerase	SV40	26085626	615
HeLa	telomerase	tumor	38688230	2165
HT1080	telomerase	tumor	34073022	2155
HT1080 hTR TTA	telomerase	tumor	32823698	9717
HT1080 hTR TCA	telomerase	tumor	35779208	3476
WI38-VA13/2RA	ALT	SV40	36972906	6842
JFCF-6/T.1R	ALT	SV40	35818652	3391
JFCF-6/T.1M	ALT	SV40	40999726	4961
GM847	ALT	SV40	67668810	13210
AG11395	ALT	SV40	32503806	3499
IIICF/c	ALT	spontaneous	71156956	6797
Saos-2	ALT	tumor	65095424	8011
U2OS	ALT	tumor	67769690	40412
SUSM-1	ALT	chemical	27673802	5293

**Figure 2.**
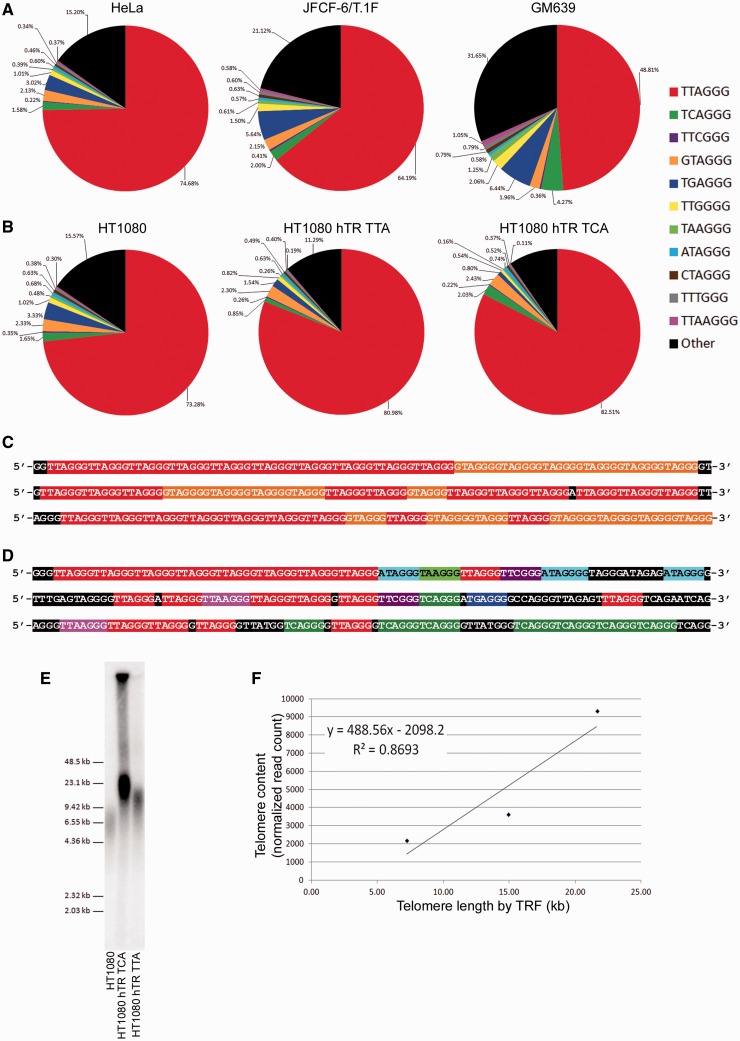
Telomerase-positive cells display telomere sequence heterogeneity. (**A**) Percentage of canonical and variant repeats in telomere reads extracted using the criterion 6× non-consecutive TNAGGG in the telomerase-positive cell lines HeLa, JFCF-6/T.1F and GM639. (**B**) Percentage of canonical and variant telomeric repeats in unmodified HT1080 cells, and in HT1080 cells with increased telomerase activity resulting from exogenous expression of wild-type hTR (HT1080 hTR TTA) or wild-type plus mutant hTR encoding the TCAGGG sequence (HT1080 hTR TCA). (**C**) Examples of 100-nt telomere reads extracted from HeLa whole-genome sequencing data demonstrating interspersion of canonical telomeric repeats with GTAGGG variant repeats. (**D**) Examples of 100-nt telomere reads extracted from HeLa whole-genome sequencing data demonstrating other sequences. (**E**) TRF analysis of telomere length in HT1080, HT1080 hTR TTA and HT1080 hTR TCA cell lines. (**F**) Correlation between telomere length measured by densitometric quantitation of TRF and telomere content measured by normalized read count using the extraction criterion 6× non-consecutive TNAGGG.

The criterion 6× non-consecutive TNAGGG was selected for all further analyses, for several reasons. First, telomere content determined by normalized read count was comparable to telomere length measured by TRF analysis (Figures 2F and 6B); second, specifying a total of six repeat reads enriches for the extraction of true telomeric reads in favor of interstitial sequences; and third, permitting non-consecutive repeats enables the extraction of other interspersed variants. TNAGGG selection also allows for the extraction of the common proximal telomere variant repeats TCAGGG and TGAGGG (13,14,28). One important caveat to the extraction criterion is that 6× non-consecutive TNAGGG will favor the extraction of position 2 variants, and may result in an underrepresentation of position 1 and 3 variants.

To determine whether whole-genome sequencing can be used to identify telomere-integrated sequences, we carried out whole-genome sequencing on the atypical ALT cell line AG11395 which contains abundant SV40 viral DNA interspersed throughout its telomeres (34,35). Interspersion is likely to have originated from a single telomeric integration of SV40 DNA, followed by the spreading of both viral and telomeric DNA sequences as a result of ALT-mediated telomere synthesis. By isolating paired end reads using the criterion 6× non-consecutive TNAGGG we identified SV40 viral DNA associating with telomere reads in the AG11395 cell line (data not shown).

In contrast to the telomeres of AG11395 cells which contain extensive amounts of SV40 sequence, GM847/Tel-2 is a previously characterized cell line which contains a limited number of telomerically integrated copies of a DNA tag that includes the neomycin resistance gene (11). To determine whether whole-genome sequencing can be used to identify low abundance sequences within telomeres, we therefore analyzed paired end reads in GM847/Tel-2 cells and in the untagged GM847 parent cells. Neomycin-resistance gene sequence was detected in telomere reads extracted from GM847/Tel-2, but not in GM847 cells (data not shown), demonstrating the ability of whole-genome sequencing to identify low abundance sequences inserted into telomeric DNA.

To validate the application of whole-genome sequencing for the analysis of telomere variant repeat content, we sequenced the parental HT1080 cell line, as well as the HT1080 cell line derivatives HT1080 hTR TTA, which expresses exogenous wild-type hTR resulting in long telomeres extended with canonical telomere sequence, and HT1080 hTR TCA, which expresses exogenous wild-type and mutant hTR resulting in long telomeres extended with both canonical and TCAGGG variant repeats. We identified both increased telomere read number and increased canonical repeat units within the extracted telomere reads in the HT1080 hTR TTA and HT1080 hTR TCA cell lines compared to the parental HT1080 cell line (Figure 1B), consistent with the telomere length increase determined by TRF analysis (Figure 2E and F). In addition, we identified increased TCAGGG variant repeat incorporation in extracted telomeric reads from the HT1080 hTR TCA cell line, compared with the HT1080 and HT1080 hTR TTA cell lines, consistent with exogenous expression of the mutant-specific hTR template (Figure 1B). This increase in TCAGGG repeat content corresponded with the observed increase in TCAGGG variant repeats detected in the HT1080 hTR TCA cell line by TRF analysis using variant and canonical repeat probes, and by telomeric-FISH using variant and canonical PNA probes (28).

To determine the contribution of sequencing error to the assignment of variant repeats, we considered the base call accuracy or quality score. The quality score measures the accuracy of the sequencing platform for an empirical data set of known accuracy, and indicates the probability that a given base is called incorrectly by the sequencer. The quality score is given as a phred value, calculated using the formula

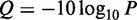

where 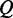 is the phred quality score and *P* is the probability that the base is incorrect. A quality score of 30 is equivalent to the probability of an incorrect base call of 1 in 1000 (or a base call accuracy of 99.9%), and is considered the benchmark for quality in whole-genome sequencing. We compared the average quality score for each hexameric repeat unit in the extracted telomere reads across the panel of cell lines (Figure 1C). Over 70% of repeats were assigned a quality score of ≥30, demonstrating a very high level of sequence accuracy in the vast majority of telomeric repeat units. Consequently, the Illumina HiSeq 2000 platform enables robust base calling of telomeric sequences, facilitating both quantitative measurement of telomere content as well as accurate analysis of telomere sequence.

### Telomerase-positive cells display telomere sequence heterogeneity

Whole-genome sequencing followed by telomere read extraction was used to investigate telomere sequence content in telomerase-positive cell lines. By normalizing the extracted reads to genome coverage, we found that the canonical telomere repeat constituted the majority of telomeric sequence reads (Figure 2A and B). It has previously been demonstrated that the proximal regions of human telomeres are rich in TCAGGG, TGAGGG and TTGGGG variant sequences (12,13), and our data confirm the relative abundance of these specific variant sequences. However, a substantial proportion and variety of other variant telomeric repeat sequences were also identified in the telomeres of telomerase-positive cell lines, including GTAGGG, ATAGGG, CTAGGG, TTCGGG, TTTGGG, TAAGGG and the 7-mer TTAAGGG (Figure 2A and B).

The GTAGGG variant repeat was surprisingly prevalent in the extracted telomeric reads. This repeat type has not previously been identified in human telomeres, and was found to cluster in blocks among canonical repeats (Figure 2C). The underrepresentation of this repeat in the proximal regions and its predominant association with canonical repeats implies it is mostly located in more distal regions of the telomere. The significant proportion of ‘other’ repeats comprised incomplete repeat units at the ends of the 100-nt sequence reads as well as other degenerate repeats or subtelomeric sequences (Figure 2D).

Telomerase uses its intrinsic hTR template region to extend the telomere terminus with canonical telomere repeats. To determine whether the proportion of variant repeats is altered following telomerase-mediated telomere extension, we analyzed telomere sequence content in the matched HT1080 telomerase-positive cell lines described in the previous section, which display varying amounts of telomerase activity and telomere lengths (Figure 2B). In addition, the HT1080 hTR TCA cell line expresses both exogenous wild-type and exogenous mutant hTR resulting in substantial telomere extension and incorporation of the TCAGGG variant repeat (28). Telomere length was measured by TRF analysis (Figure 2E), and found to correlate with normalized telomere content determined by the number of telomere reads extracted from the whole-genome sequencing data (Figure 2F).

An increase in TCAGGG variant repeat content was observed in the HT1080 hTR TCA cell line, consistent with telomerase-mediated incorporation of mutant TCAGGG repeats using the exogenous mutant template (Figure 2B). Notably, the proportion of some variant repeats remained similar following telomerase-mediated extension, in contrast to the decreased proportion that would be predicted following synthesis of large tracts of telomeric DNA containing only canonical repeats (Figure 2B). One possible explanation for this may be that telomerase is error-prone, so variant repeats are generated by telomerase-mediated telomere extension.

### Telomerase-mediated telomere extension results in biased variant repeat synthesis

**Figure 3.**
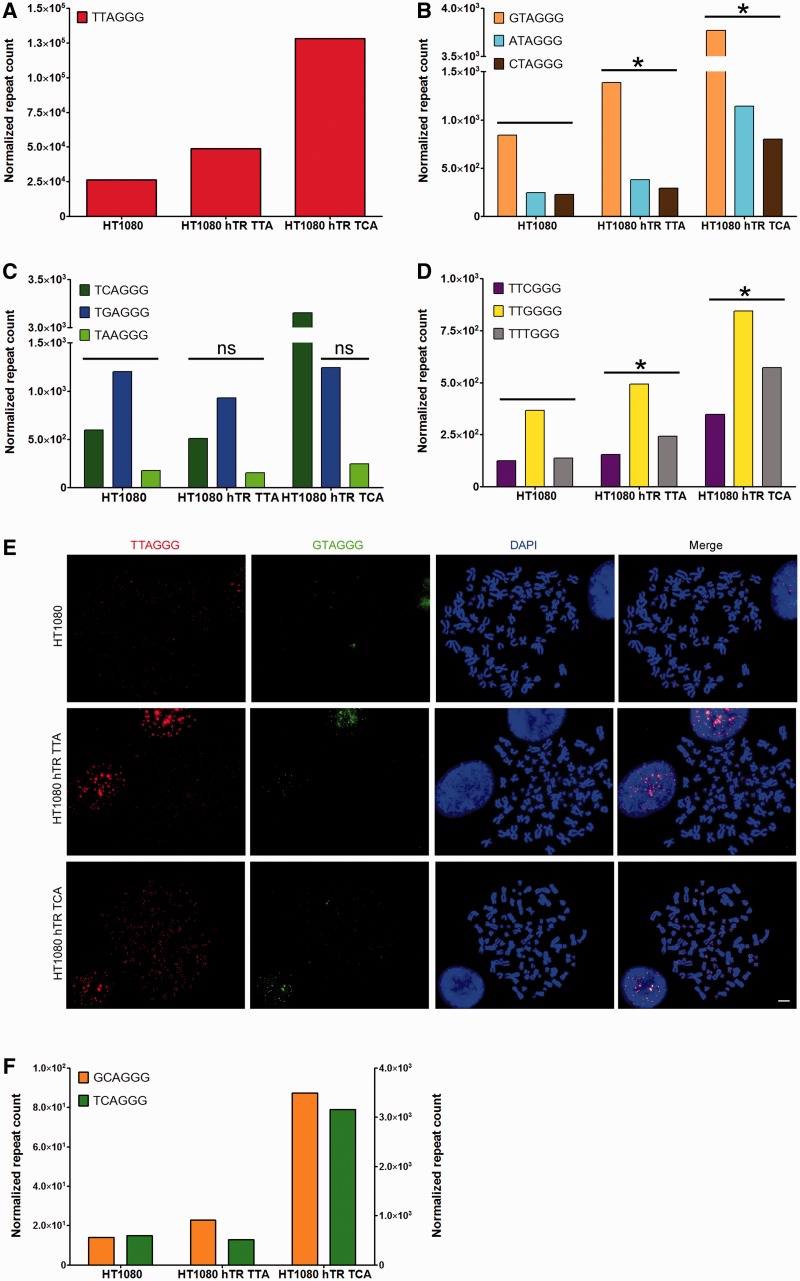
Telomerase overexpression results in telomere lengthening and biased variant repeat generation. (**A**–**D**) Telomere and variant repeat count in telomere extracted reads using the criterion 6× non-consecutive TNAGGG normalized to genome coverage in the HT1080 parental, HT1080 hTR TTA (expressing exogenous wild-type hTR) and HT1080 hTR TCA (expressing exogenous wild-type and mutant hTR) cell lines. The statistical significance was determined by calculating the probability that the increase in variant repeats arose due to a single base error in sequencing for each site. Asterisk represents *P* < 0.001, ns represents not significant. The TCAGGG variant was removed from statistical analysis in the HT1080 hTR TCA cell line. (**E**) Telomeric localization of GTAGGG repeats by telomere (red) and variant repeat (green) FISH on metaphase spreads, counterstained with DAPI. Bar represents 5 µm. (**F**) GCAGGG variant repeat count (orange; left axis) and TCAGGG variant repeat count (green; right axis) in telomere extracted reads from matched HT1080 cell lines.

To further investigate the generation of variant repeats in telomerase-positive cells, we categorized variant repeats by the specific site within the hexameric repeat and quantitated the number of each nucleotide at each position in the telomere reads extracted from HT1080, HT1080 hTR TTA and HT1080 hTR TCA cells. Variant telomeric repeats involving positions 4–6 were scarce, so we focused our analysis on hexameric nucleotide positions 1–3. Consistent with telomere length, the number of canonical repeats present in the extracted telomeric reads increased in the HT1080 hTR TTA and to a greater extent in the HT1080 hTR TCA cells compared to the parental HT1080 control (Figure 3A).

The total content of variant repeats involving nucleotide position 1 of the hexameric repeat (GTAGGG, ATAGGG and CTAGGG) increased as a function of telomere length in the cells with increased telomerase activity (Figure 3B). No increase in TGAGGG or TAAGGG variant repeats was observed following telomere extension, however an increase in the TCAGGG variant repeat was observed with exogenous overexpression of the mutant hTR (Figure 3C). We also identified a telomere length-dependent increase in TTCGGG, TTGGGG and TTTGGG variant repeats, which differs from the canonical repeat at position 3 of the hexameric unit (Figure 3D). This indicates that variants at hexameric nucleotide position 2 are not associated with telomerase-mediated telomere extension.

Variant repeat misrepresentation by sequencing error was excluded by the site-biased increase in variant repeats at positions 1 and 3, but not position 2 of the hexameric repeat unit, being significantly greater than the sequencing error rate corresponding to a quality score of 30. Illumina sequencing chemistry reportedly delivers the majority of bases with a quality score of ≥30, as demonstrated by the average repeat unit quality scores for the extracted telomere reads (Figure 1C). The increase in GTAGGG variant repeats was most striking, and was also demonstrated by canonical and variant repeat FISH analysis on cytocentrifuged chromosomes (Figure 3E).

To further corroborate the biased synthesis of variant repeats, we identified a significant increase in GCAGGG variants, specific to the HT1080 hTR TCA cell line (Figure 3F). This variant repeat is detected at very low frequency in all other cell lines, but appears to be generated by the mutant hTR template as a function of telomere length, presumably by a similar mechanism to GTAGGG variant repeat synthesis by the wild-type hTR template. These data demonstrate that variant repeats that differ at nucleotide positions 1 and 3 of the hexameric repeat unit are generated in telomerase-positive cells at a rate proportional to that of canonical telomere repeats, indicating that variant repeats are most likely synthesized during telomerase-mediated telomere extension.

### Telomere sequence heterogeneity exists in normal human cells

In order to investigate telomere sequence content in the absence of a TMM, we analyzed canonical and variant repeat content in extracted telomere reads from normal human fibroblast cells. We reasoned that, despite the absence of an active TMM, normal human cells are all derived from germline cells which have undergone telomere length resetting by telomerase. Consequently, the telomere sequence of mortal human cells will reflect, at least in part, the normal activity of telomerase.

**Figure 4.**
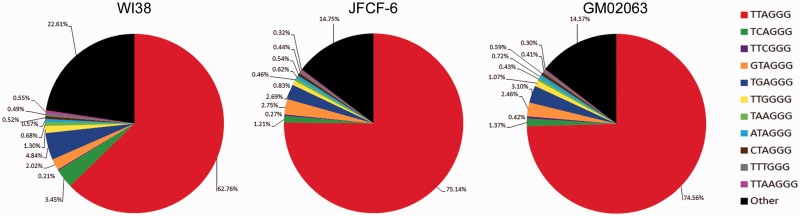
Normal human cells display telomere sequence heterogeneity. Percentage of canonical and variant repeats in telomere reads extracted using the criterion 6× non-consecutive TNAGGG in the mortal human cell strains WI38, JFCF-6 and GM02063.

Analysis of telomere sequence content in the WI38, JFCF-6 and GM02063 mortal human cell strains revealed extensive sequence heterogeneity (Figure 4). As expected, the canonical repeat dominated. Variant repeats that exist in the proximal regions of the telomere, specifically TCAGGG, TGAGGG and TTGGGG were most abundant (13,14). Similarly to the telomerase-positive cell lines, we observed a relatively high proportion of GTAGGG and ATAGGG variant repeats. These repeats do not substantially contribute to the proximal regions of telomeres (13,14), and are therefore most likely to occur in more distal telomeric locations. This is consistent with an association between sequence-biased variant repeat generation and telomerase-mediated telomere lengthening in non-tumorigenic, normal human cells.

### ALT-mediated telomere extension results in stochastic variant repeat synthesis

**Figure 5.**
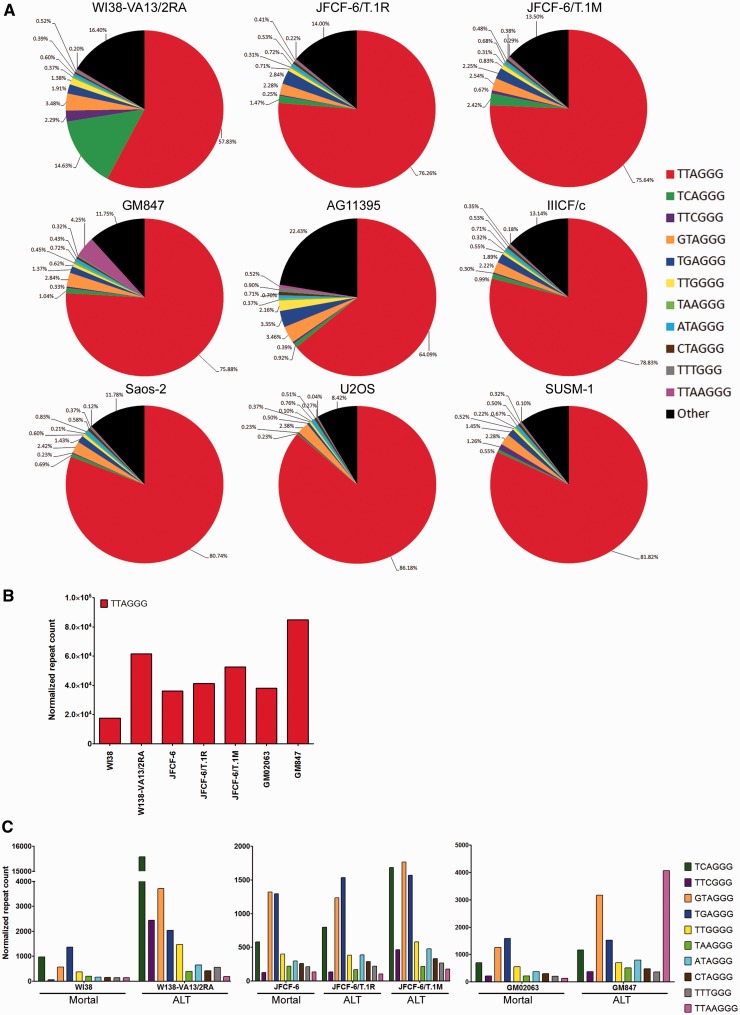
ALT cells display telomere and variant repeat amplification. (**A**) Percentage of canonical and variant repeats in telomere reads extracted using the criterion 6× non-consecutive TNAGGG in the ALT cell lines WI38-VA13/2RA, JFCF-6/T.1R, JFCF-6/T.1M, GM847, AG11395, IIICF/c, Saos-2, U2OS and SUSM-1. (**B**) Telomere repeat count in extracted telomere reads from matched mortal and ALT cell lines (WI38 and WI38-VA13/2RA, JFCF-6 and JFCF-6/T.1R and JFCF-6/T.1M, GM02063 and GM847). (**C**) Variant repeat count in extracted telomere reads from matched mortal and ALT cell lines (WI38 and WI38-VA13/2RA, JFCF-6 and JFCF-6/T.1R and JFCF-6/T.1M, GM02063 and GM847).

We have previously demonstrated that the SV40-immortalized WI38-VA13/2RA ALT cell line contains an abundance of TCAGGG variant repeats (28). Our data suggest that the TCAGGG variant repeat, which involves a nucleotide change at position 2 of the hexameric repeat unit, is not generated by telomerase (Figure 3C). To determine whether TCAGGG variant repeat abundance is characteristic of the ALT mechanism and, more generally, whether ALT cells display a variant repeat profile that is distinct from telomerase-positive and mortal cells, we extracted telomere sequences using the same criterion as for normal and telomerase-positive libraries in a panel of nine ALT cell lines (Figure 5A). Telomere repeat content was normalized to genome coverage.

We found that the ratio of total variant repeats to canonical sequences in ALT cells was indistinguishable from that observed in telomerase-positive and mortal cells. However, ALT cell lines were characterized by very substantial variation in variant repeat types. For example, as reported previously, the WI38-VA13/2RA cell line contained a substantial amount of the TCAGGG variant repeat (28), whereas the GM847 line was the only ALT line in this panel that had abundant TTAAGGG 7-mer repeats (Figure 5). AG11395 cells contained an abundance of other sequences, including SV40 viral DNA, in their telomeres (data not shown) (34,35). The cell line-specific content of certain variant repeats or viral DNA is consistent with the previously proposed model of recombination-mediated interspersion of variant repeats which are copied in a stochastic manner from other telomeric regions (36).

Cells that use the ALT mechanism of telomere maintenance typically have telomeres that are highly heterogeneous in length but long on average, as well as abundant extrachromosomal telomeric DNA, and therefore a high total telomeric DNA content. By comparing mortal cell strains with ALT cell lines that were derived from them, we demonstrated an increase in canonical telomere repeat count in the ALT cell lines WI38-VA13/2RA, JFCF-6/T.1R, JFCF-6/T.1M and GM847, compared with the mortal precursor cell strains WI38, JFCF-6 and GM02063 (Figure 5B). Given that the proportion of variant repeats in ALT cells was similar to that of non-ALT cells, the increased telomeric content in ALT cells means that the total amount of variant repeats must have increased. As expected therefore, the normalized repeat count showed an increase in all variant repeats in the immortal ALT cell lines including variants commonly found in the proximal region of the telomere, but with some variants increasing more than others in individual ALT lines (Figure 5C). This indicates that the increase in variant repeats in ALT cells overall does not display a sequence bias, and suggests that there is some randomness in the extent to which specific variant sequences become amplified by the ALT mechanism. This contrasts with the biased variant repeat synthesis that accompanies telomerase-mediated telomere extension.

### Telomere length analysis by whole-genome sequencing

Whole-genome sequencing provides a measurement of telomere repeat content (37), and has recently been used to characterize changes in telomeric DNA during malignant progression of pediatric cancers (33). Telomeric content measured by quantitating sequence reads containing four consecutive TTAGGG repeats normalized to whole-genome coverage has previously been validated by TRF length analysis, telomere FISH on interphase nuclei and by qPCR (33). Here, we examined whether the presence of variant repeats throughout the telomere repeat array is likely to impact upon telomere length measurement techniques.

**Figure 6.**
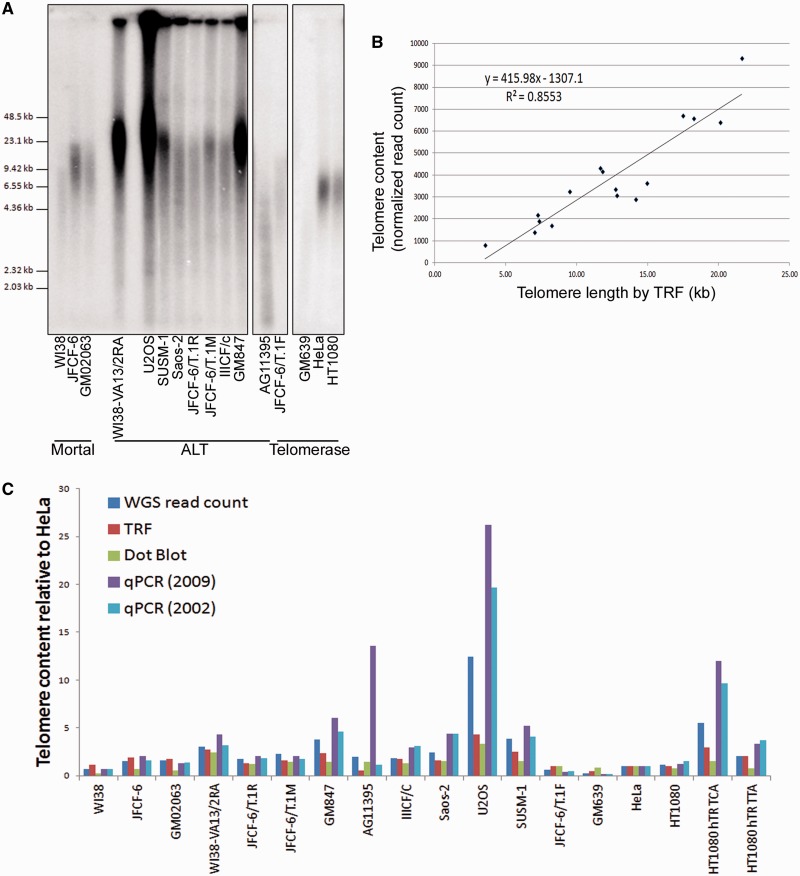
Whole-genome sequencing provides an accurate measurement of telomeric content. (**A**) TRF analysis of telomere length in mortal, ALT and telomerase-positive cell lines. (**B**) Correlation between telomere length measured by densitometric quantitation of TRF and telomere content measured by normalized read count in mortal, ALT and telomerase cell lines. AG11395 and U2OS were excluded from this analysis due to inaccuracies in densitometric TRF quantitation caused by telomeric SV40 sequences in AG11395 and exceptionally long telomeres in U2OS. (**C**) Comparison of telomere repeat content measured by normalized telomere read count (4× consecutive TTAGGG), TRF analysis, dot blot, qPCR using 2009 published primers (31) and qPCR using 2002 published primers (32), relative to the HeLa cell line.

To determine whether whole-genome sequencing can be accurately applied to the measurement of telomere length in cells with different TMMs, substantially different telomere lengths, and different variant repeat sequence contents, we compared telomere content measured by whole-genome sequencing using 4× consecutive TTAGGG repeats as the read extraction criterion, to telomere length measured by TRF analysis and to telomere content measured by dot blot and by qPCR. Telomere length analysis by TRF (Figure 6A) was quantitated by densitometry and found to correlate well (*r*^2^ = 0.855) with telomere sequence read counts normalized to genome coverage (Figure 6B). The cell lines AG11395 and U2OS were excluded from this direct comparison due to inaccuracies in densitometric TRF quantitation caused by telomeric SV40 sequences in the AG11395 cell line and by the exceptionally long telomeres present in the U2OS cell line. The intercept of the line graph is negative, which reflects technical differences that arise when comparing telomere sequence content to TRFs which include variable amounts of subtelomeric regions dictated by the terminal restriction enzyme site.

The correlation with length measurements by the other two techniques was generally good, but there were some substantial discrepancies (Figure 6C). The most striking anomalies arose using the qPCR technique, specifically in the ALT cell lines and in the HT1080 hTR TCA variant repeat cell line (Figure 6C). In order to generate fixed length PCR products, the 2009 multiplexed qPCR method of telomere length measurement utilizes a terminal 3′ mismatch in the reverse primer, which can only extend forward primer extension products (31). The presence of variant repeats within telomeres means that there is the potential for this ‘mismatched’ primer to match some telomeric DNA sequences. We quantitated the number of amplification-competent reverse primer sites [TGTTAGGG(NTAGGG)_4_NTA] in the extracted telomere reads from all cell lines, and found the number of reverse primer sites in individual cell lines accounts for the instances where telomere content is overestimated by qPCR compared to the other techniques (Figure 6C, and data not shown). This overestimation was reduced by using the 2002 previously established qPCR primers that do not utilize a sequence mismatch in the reverse primer (32) (Figure 6C). This provides a caveat to the 2009 multiplexed qPCR method of telomere length quantitation, which does not take into account the presence and in some cell types, abundance, of variant telomeric sequences.

## DISCUSSION

We have analyzed telomere sequence content in a panel of mortal, telomerase-positive and ALT cell lines, and identified extensive sequence divergence within human telomeres. Many different variant sequences were detected, predominantly involving a single nucleotide alteration within the first 3 nt of the TTAGGG hexameric repeat unit. In contrast, the GGG motif was mostly invariant, which suggests that there is a selection pressure to retain this sequence. The most common variant repeats were TCAGGG, TGAGGG and GTAGGG. The first two of these as well as TTGGGG variant repeats have previously been found to be common in the proximal regions of human telomeres (12,13).

Our data demonstrate that telomerase-mediated telomere extension is accompanied by generation of variant repeats, specifically involving a base change at the first nucleotide (GTAGGG, ATAGGG and CTAGGG), and the third nucleotide (TTCGGG, TTGGGG, TTTGGG), but not at the second nucleotide (TCAGGG, TGAGGG, TAAGGG) of the hexameric repeat unit. Variant repeat misrepresentation by sequencing error was excluded by the site-biased increase in variant repeats being significantly greater than the 1 in 1000 sequencing error rate corresponding to a quality score of 30. The mechanism responsible for the observed bias in variant telomere repeat generation in telomerase-positive cells is not known. It is possible that the entire telomere may be susceptible to intra-telomeric replication errors such as base mismatches and replication slippage events that have been documented to occur at low frequency in normal human somatic cells (38). It is also possible that oxidative stress during prolonged *in vitro* culturing may influence telomere sequence, although it must be noted that these sequences were also observed in HT1080 cells where the telomeres had recently been extended by overexpression of exogenous hTR. While inter-telomeric templating of variant repeats cannot be ruled out, no copying of a telomerically-integrated neomycin tag was detected in these telomerase-positive cell lines (28,39). Template-independent terminal transferase activity of telomerase may also offer a partial explanation for variant repeat synthesis (40). However, none of these mechanisms adequately explain the observed sequence bias in the telomerase-generated variant repeats.

A more plausible explanation is misincorporation by telomerase. It has previously been shown that DNA synthesis by the HIV-1 reverse transcriptase is inaccurate and prone to mutational hotspots (41). Furthermore, yeast telomerase generates species-specific variant repeats (42). The enzyme kinetics of telomerase-mediated extension, telomerase pausing and telomerase processivity are not fully understood and it is possible that variations in enzyme stability at specific template positions, or an induced-fit mechanism of telomere extension, combined with a lack of proof-reading capabilities, may result in misincorporation at the first and third nucleotide of the telomere hexamer. Misincorporation by telomerase would result in the *de novo* synthesis of variant repeats directly at the telomere terminus, and the combined addition of canonical and variant sequences throughout the extended region of the telomere. This finding is relevant to telomere capping function and the formation of telomere secondary structures, which are sequence-dependent.

The GTAGGG variant repeat was common in all cell lines analyzed, and its content increased most substantially following telomerase-mediated telomere extension. The GTAGGG variant is not common in the proximal regions of human telomeres, the sequence of which has been previously documented (13–15) and analysis of extracted telomeric reads identified GTAGGG repeats predominantly among blocks of canonical repeats. This suggests that GTAGGG repeats occur at more distal locations within the telomere, which have to date evaded sequencing. A relative increase in GCAGGG compared with TCAGGG variant repeats was observed specifically in the HT1080 hTR TCA cell line, further implicating misincorporation by telomerase as the most likely mechanism of telomerase-associated variant repeat synthesis.

A general increase in the amount of variant repeats was identified in ALT cells, regardless of whether the variants are usually found in the proximal telomere. This is consistent with telomere synthesis by recombination-mediated telomere templating, which can be initiated anywhere within the telomere repeat array, including the variant repeat-rich proximal regions (38). We have previously shown that the WI38-VA13/2RA ALT cell line contains a substantial proportion of TCAGGG variant repeats. In the present study we found that this is not typical of all ALT cell lines, and variant repeat content appears to be cell line specific, consistent with stochastic amplification of telomeric regions. By comparing matched mortal cell strains and immortal ALT cell lines overall, no sequence bias was detected in the amplified variant repeats. This indicates the existence of mechanistically distinct processes of variant repeat synthesis associated with telomerase- and ALT-mediated telomere extension.

We have previously proposed that elevated variant repeats in ALT cells displace shelterin, facilitating telomeric recombination, and resulting in a self-perpetuating change in telomeric sequence that ‘locks in’ ongoing telomere length maintenance by ALT activity. In this more extensive study of telomere sequence content in multiple cell lines, we did not observe an increase in the proportion of total variant repeat content in all ALT cell lines. However, ALT cells display considerably longer and more heterogeneous telomere lengths, and the total amount of variant repeats increases concomitantly. This increase in variant repeat content and its heterogeneous distribution within telomeres may contribute to shelterin displacement, altering the balance of telomere capping function, and thus enabling telomeric recombination.

Moreover, the variant repeat sequences that are present in ALT cells differ from those in telomerase-positive cells, which may have functional consequences. For example, TCAGGG is a variant repeat that increases in frequency in ALT but not telomerase-positive cell lines, and we have demonstrated both *in vitro* and *in vivo* that a group of nuclear receptors, which bind with high affinity to this repeat type, competitively displaces shelterin and that introduction of this sequence into the telomeres of telomerase-positive cells results in acquisition of some ALT-like characteristics (28).

Interestingly, variant repeats in telomerase-positive cells are also likely to result in decreased shelterin binding. The shelterin complex binds specifically to canonical TTAGGG repeats, and it has been shown *in vitro* that TRF1 and TRF2 binding is diminished by the CTAGGG variant repeat (16) and abolished by TTGGGG (43,44). Furthermore, variant repeats may affect telomeric higher order structures. For instance, the GTAGGG variant was detected in small clusters, which are predicted to form Hoogsteen hydrogen-bonded G-quadruplex structures with higher stability than the canonical telomere repeat, because of the extra guanine residue. Consequently, variant repeats have the potential to substantially alter the telomere nucleoprotein structure through specific sequence–protein interactions and by the formation of DNA secondary structures that depend on the variant repeat landscape. It is also possible that depletion of shelterin binding to variant repeats may expose telomeres to replication fork stalling, resulting in telomere fragility.

In summary, variant repeats are more prevalent at human telomeres than previously appreciated, and are produced by mechanistically distinct processes during telomerase- and ALT-mediated telomere extension. There is evidence that telomerase may misincorporate nucleotides at specific positions on its RNA template; this warrants further investigation and may provide insight into the mechanism of telomerase extension and processivity. The functional consequences of variant repeats within telomeres most likely include decreased shelterin binding and altered higher order structure, and may be profound.

## FUNDING

National Health and Medical Research Council project grant [#1009231 to R.R.R. and H.A.P,]; Cancer Council New South Wales program grant (to R.R.R.); Cancer Institute New South Wales fellowship (to H.A.P.). Funding for open access charges: Children's Medical Research Institute, 214 Hawkesbury Road, Westmead NSW 2145 Australia.

*Conflict of interest statement*. None declared.
